# A Peptide-Based Virus Inactivator Protects Male Mice Against Zika Virus-Induced Damage of Testicular Tissue

**DOI:** 10.3389/fmicb.2019.02250

**Published:** 2019-09-27

**Authors:** Lulu Si, Yu Meng, Fang Tian, Weihua Li, Peng Zou, Qian Wang, Wei Xu, Yuzhu Wang, Minjie Xia, Jingying Hu, Shibo Jiang, Lu Lu

**Affiliations:** ^1^Key Laboratory of Medical Molecular Virology (MOE/NHC/CAMS), School of Basic Medical Sciences and Shanghai Public Health Clinical Center, Fudan University, Shanghai, China; ^2^NHC Key Laboratory of Reproduction Regulation (Shanghai Institute of Planned Parenthood Research), Fudan University, Shanghai, China; ^3^Lindsley F. Kimball Research Institute, New York Blood Center, New York, NY, United States

**Keywords:** inactivator, peptide, Zika virus, testicular tissue damage, sperm

## Abstract

Zika virus (ZIKV) was a re-emerging arbovirus associated with Guillain–Barré Syndrome in adult and congenital Zika syndrome in fetus and infant. Although ZIKV was mainly transmitted by mosquito bites, many sexual transmission cases have been reported since the outbreak in 2015. ZIKV can persist in testis and semen for a long time, causing testicular tissue damage and reducing sperm quality. However, no drug has been approved for prevention or treatment of ZIKV infection, especially infection in male testicular tissue. Previously reported peptide Z2 could inactivate ZIKV, inhibiting ZIKV infection *in vitro* and *in vivo*. Importantly, Z2 could inhibit vertical transmission of ZIKV in pregnant mice, reducing ZIKV infection in fetus. Here we showed that intraperitoneally administered Z2 could also be distributed to testis and epididymis, resulting in the reduction of ZIKV RNA copies in testicular tissue and protection of testis and epididymis against ZIKV-induced pathological damage and poor sperm quality in type I interferon receptor-deficient A129 mice. Thus, Z2, a ZIKV inactivator, could serve as an antiviral agent for treatment of ZIKV infection and attenuation of ZIKV-induced testicular tissue damage.

## Introduction

Zika virus was a re-emerging arbovirus (Gao, 2018; Pettersson and Bohlin, 2018), like dengue virus (DENV) and Japanese encephalitis virus (JEV), belonging to *Flavivirus* genus in the *Flaviviridae* family. Since the outbreak in Brazil in 2015, ZIKV has rapidly spread to 87 countries and territories^1^ (Baud et al., 2017a; Gorshkov et al., 2018), attracting global attention. About 80% of those infected with ZIKV presented with asymptomatic, or only mild illness (Petersen E. et al., 2016; Pierson and Diamond, 2018). However, ZIKV infection has been associated with more severe complications: Guillain–Barré Syndrome in adult (Brasil et al., 2016; Peixoto et al., 2019) and congenital Zika syndrome in fetus and infant (Baud et al., 2017b; Gurung et al., 2019).

ZIKV is mainly transmitted through mosquito bites (Petersen L.R. et al., 2016), while it can also be transmitted via *in utero* from mother to fetus (Besnard et al., 2014), blood transfusion (Tai et al., 2019) and intercourse (Duggal et al., 2017; Mead et al., 2018; Sakkas et al., 2018). It is reported that ZIKV was transmitted through sexual contact, perhaps up to 41 days after the onset of symptoms (Turmel et al., 2016), and infective virions were still isolated from semen 69 days after infection (Arsuaga et al., 2016; Garcia-Bujalance et al., 2017). The viral load in semen was 100,000 times that in the blood at 2 weeks post-infection (Mansuy et al., 2016), and viral RNA was still detected up to 370 days after illness onset (Barzon et al., 2018). It is also reported that ZIKV infection caused patients to have a decreasing total sperm count in the acute phase of infection (Joguet et al., 2017) and abnormal spermogram results 1 year after infection (Avelino-Silva et al., 2018), suggesting ZIKV was harmful to human spermatozoa production. Testis explants from uninfected donors were also proven to be susceptible to ZIKV infection (Matusali et al., 2018). As determined from an *in vitro* human testicular organoid culture system, ZIKV-infected testicular organoids may lead to multiple kinds of cell death (Strange et al., 2018). Although little was known about ZIKV infection in human testis and epididymis, except for semen, many murine models were used to study damage to testicular tissue. Govero et al. (2016) performed a study in wild-type C57BL/6 mice in the presence of the anti-Ifnar1 antibody and revealed that ZIKV preferentially infected spermatogonia and Sertoli cells in the testis. This led to cell death and destruction of the seminiferous tubules in association with testis damage and poor sperm quality (Govero et al., 2016). Ma et al. (2017) also established a mouse model using IFNα/β receptor-deficient mice (*Ifnar1^–/–^* knockout mice), and demonstrated that ZIKV infection induced inflammation in the testis and epididymis, leading to severe damage to testes at 60 days post-infection. Taken together, these findings suggested that ZIKV could persist in testicular tissue for a long time, causing severe damage to testis and epididymis and reducing sperm quality.

**FIGURE 1:**
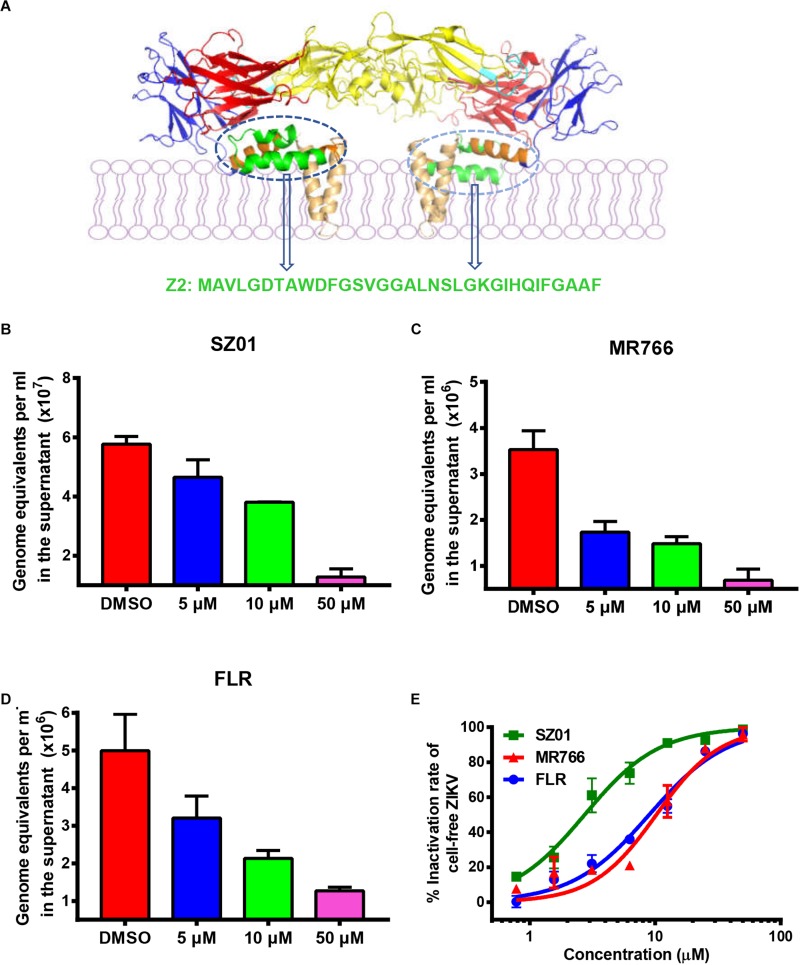
Z2 inhibited infection of ZIKV strains with Asian and African lineages in TM4 cells. **(A)** Sequence and location of Z2 in ZIKV E dimer. Side view of ZIKV E protein in the dimeric conformation is shown [Protein Data Bank: 5IRE (Sirohi et al., 2016)]; DI, DII, DIII, stem and transmembrane domain are shown in red, yellow, blue, orange, and wheat, respectively; the fusion peptide is shown in cyan, and Z2 (421–453) is shown in green. TM4 cells were infected with ZIKV strain SZ01 **(B)**, MR766 **(C)** and FLR **(D)**, treated with Z2 at different concentration, and then viral copies in the supernatant at 48 h were measured by qRT-PCR. Data were presented as means ± SD. **(E)** Z2-treated ZIKV of different strain lost infectivity on TM4 cells irreversibly. After incubation with Z2 at 37°C for 2 h, ZIKV particles were separated from the unbound Z2 by PEG 8000 to measure their infectivity on TM4 cells. Each sample was tested in triplicate and the experiments were repeated at least once. The data from two independent experiments were presented as mean ± SD.

Currently, no approved drug is available to inhibit ZIKV infection (da Silva et al., 2018), especially infection in testicular tissue. Ebselen (EBS), an antioxidant in clinical trials, was reported to alleviate testicular pathology in ZIKV-infected mice by reducing the level of oxidative stress and proinflammatory cytokines. However, it only had a weak effect on ZIKV directly, and its safety for pregnant women was unknown (Simanjuntak et al., 2018). This calls for the development of safe and effective drugs to prevent ZIKV-induced testicular damage. The testis is a male reproductive organ, mainly producing spermatozoa and androgen. Specifically, spermatogenesis is a complex cellular event taking place in the seminiferous epithelium of seminiferous tubules and protected by Sertoli cells that form the blood–testes barrier (BTB) by tight junction protein (Su et al., 2011). The BTB provides a specialized microenvironment for spermatogenesis by preventing harmful agents from entering the seminiferous tubule, but this was found to pose a major obstacle to the delivery of therapeutic drugs to the seminiferous epithelium (Cheng and Mruk, 2012). Therefore, any drug able to prevent ZIKV-induced damage in testicular tissue should be able to cross the BTB into seminiferous tubules, or reach the testicular tissue, to inhibit ZIKV from entering into seminiferous tubules. The most promising anti-ZIKV drugs so far include small-molecule compounds (Deng et al., 2016a; Chan et al., 2017; Li et al., 2017), antibodies (Zhang et al., 2016; Wang et al., 2017, 2019), and peptides (Yu et al., 2017; Jackman et al., 2018). Compared with small-molecule compounds, peptides were safer, especially for pregnant women. As a macromolecular substance, passing through the BTB was challenging for antibodies, and it is reported that the concentration of specific IgG entering into the rete testis was 0.6% of that in blood serum (Knee et al., 2005). Therefore, the safer and cheaper peptide drugs, which consisted of dozens of amino acids, began to gain gradual acceptance. We previously demonstrated that an amphipathic peptide Z2, derived from the stem region of ZIKV E protein (Figure 1A), inhibited ZIKV infection *in vivo* and *in vitro*, suggesting its promise as an anti-ZIKV candidate drug. What’s more, Z2 had a protective effect in pregnant mice and their fetuses, suggesting it was able to cross the placental barrier (Yu et al., 2017). However, whether Z2 could enter the seminiferous tubules and protect testicular tissue against ZIKV infection remained unknown.

In this work, we showed that intraperitoneally injected Z2 could be distributed in the testicular tissue. It then inhibited ZIKV infection, resulting in significant reduction of viral loads and protection of testis, epididymis, and sperm from ZIKV-induced pathological damage. These results suggest that Z2 has the potential to be further developed as an anti-ZIKV agent for treatment and prevention of ZIKV-induced damage in testicular tissue.

## Materials and Methods

### Ethics Statement and Mice

All animal experiments were carried out according to ethical guidelines and approval by Shanghai Public Health Clinical Center Animal Welfare and Ethics Committee (2016-A021-01) and Institutional Laboratory Animal Care and Use Committee at Fudan University (20160927-2). The type I interferon receptor-deficient mice A129 (male, 6–7 weeks old) were bred at the Animal Experiment Department of Shanghai Public Health Clinical Center. Experiments using A129 mice infected by ZIKV were conducted in a Biosafety Level 2 (BSL2) facility at Shanghai Public Health Clinical Center. Specific pathogen-free BALB/c mice (male, 6–7 weeks old) were bought from Shanghai Lingchang BioTech, Co., Ltd. (Shanghai, China) and bred at the Department of Laboratory Animal Science of Fudan University.

### Cells, Viruses, and Peptides

Baby Hamster Kidney (BHK-21) cells and Cercopithecus Aethiops Kidney (Vero-E6) cells were obtained from ATCC (Manassas, VA, United States). Cells were cultured in Dulbecco’s modified Eagle’s medium (DMEM, meilunbio, Shanghai, China) supplemented with 10% fetal bovine serum (FBS) at 37°C and 5% CO_2_. Mouse Testis Sertoli (TM4) cells were purchased from Zhong Qiao Xin Zhou BioTech, Co., Ltd. (Shanghai, China) and cultured in Dulbecco’s modified Eagle’s medium/Nutrient Mixture F-12 (DMEM/F12, Thermo Fisher Scientific, Waltham, MA, United States) supplemented with 5% Horse Serum and 2.5% FBS at 37°C and 5% CO_2_.

Zika virus (ZIKV) strain SZ01/2016 (GenBank No. KU866423) was kindly provided by Dr. Cheng-Feng Qin (Deng et al., 2016b) and preserved in our laboratory. ZIKV strains MR766 (#VR1838) and FLR (#VR1844) were obtained from ATCC. ZIKV was propagated in Vero-E6 cells. Briefly, Vero-E6 cells were infected with the virus at multiplicity of infection (MOI) of 0.01. The supernatants were harvested at 5 days post-infection, centrifuged at 4,000 rpm for 30 min to remove cellular debris, and stored at −80°C as stock.

Peptides Z2 (MAVLGDTAWDFGSVGGALNSLGKGIHQIFG AAF), Z2-Cy5 and scrambled peptide (LDIIAGLSAGFQ GGATFVDAHGMVKASFLGGNW) were synthesized at Kangbei Bio, Co., Ltd. (Ningbo, China) with 95% purity. Peptides were solubilized in dimethyl sulfoxide (DMSO) at 10 mM and stored at −20°C.

### Plaque Forming Assay

Virus titer was detected by plaque forming assay as shown below. BHK-21 cells were seeded onto a 12-well plate with 2 × 10^5^ cells per well and cultured overnight at 37°C and 5% CO_2_. Serially 10-fold diluted virus were added to each well and incubated for 2 h at 37°C. Then the supernatant was replaced with 1 ml DMEM containing 1% low melting agarose and 2% FBS. After agarose solidification, the cells were cultured at 37°C and 5% CO_2_ for 6 days. Then cells were fixed with 4% formalin and stained with 1% crystal violet overnight. Finally, the plaque forming units were counted, and virus titer was calculated.

### *In vivo* Small Animal Optical Imaging

Six male A129 mice (7–8 weeks old) were randomly divided into two groups. Mice in each group (*n* = 3) were injected intraperitoneally with Z2-Cy5 (100 μg in 100 μl PBS) or PBS (vehicle in 100 μl PBS) as control. After anesthetization with pentobarbital sodium, mice were imaged by the IVIS Lumina K series III *in vivo* imaging system from PerkinElmer (Waltham, MA, United States) for 1 h. To determine the distribution of Z2-Cy5 in the testicular tissue, mice were sacrificed using pentobarbital sodium, and all testes and epididymides were removed for imaging. The radiant efficiency (ps^–1^cm^–2^sr^–1^) (μW^–1^cm^–2^) in mouse body and testis and epididymis was calculated by Living Image 4.5.5 software.

### Assay for Assessing Protective Effect of Z2 Against ZIKV Infection in Testicular Tissue of Male A129 Mice

To determine the protection of Z2 against ZIKV-induced testis damage, male A129 mice (7–8 weeks old) were randomly divided into three groups (*n* = 5): Z2-treated group, vehicle-treated group, and mock-infected group. Mice in the Z2- or vehicle-treated group were intraperitoneally injected (i.p.) with 200 PFU ZIKV with Z2 (15 mg/kg) or vehicle on day 0, followed by i.p. administration of Z2 (15 mg/kg) or vehicle once daily for six consecutive days, respectively. Mice in the mock-infected group only received PBS as normal control. The body weight was monitored daily for 14 days, and blood was collected at 1, 3, 7, 11, and 16 days post-infection (d.p.i.) for detection of ZIKV copies in sera. At 16 d.p.i., mice were euthanized by CO_2_ inhalation, and the testes and epididymides were removed. The weight and size of testes were measured as previously described (de La Vega et al., 2018). After imaging, the left testes and epididymides were immersed in Bouin’s for hematoxylin-eosin (H&E) staining. The right testes and epididymides were soaked in RNAiso Plus reagent at −80°C for further detection of ZIKV copies.

### Safety Analysis of Z2 in Testicular Tissue of Male BALB/c Mice

For safety analysis of Z2 in testicular tissue, 10 male BALB/c mice (7–8 weeks old) were randomly divided into two groups. Five mice in each group were i.v. administered with Z2 at 100 mg/kg of body weight or PBS control for 3 days. The body weight of mice was monitored every other day for 30 days. Blood was collected at 4 h, as well as 1, 14, and 30 days post-injection and sera were separated from the blood samples for use in ELISA to detect the concentration of testosterone and inhibin B as well as the titer of Z2-specific antibody. At 30 days, mice were sacrificed, and the testes and epididymides were removed for histological examination.

### Computer-Assisted Sperm Analysis (CASA)

Mature sperm in the cauda epididymis of the three groups of male A129 mice were collected and placed in 1 ml PBS (preincubated at 37°C) immediately after euthanasia. The sperm suspension was analyzed for total sperm count and motility by computer-assisted sperm analysis (CASA), as previously described (Goodson et al., 2011), using Hamilton Thorne IVOS II (Beverly, MA, United States). Then, after smear, desiccation and fixation, remaining sperm were stained by the Papanicolaou staining method for manual morphological analysis. Sperm morphology was observed in each mouse.

### qRT-PCR for Detection of ZIKV RNA

ZIKV-infected mice were euthanized at 16 days post-infection. Testes and epididymides were homogenized with beads in 1 ml RNAiso Plus reagent (TaKaRa, Japan) using Tissuelyser-48 (Jingxin, Shanghai, China) after weighing. Homogenized tissue were centrifuged for 15 min at 14,000 rpm at 4°C, then total RNA in tissues was extracted according to the operating manual and stored at −80°C for the next step. Sperm collected in PBS were placed in RNAiso to extract total RNA under the same procedure. Viral RNA in sera samples on specific days was extracted using the EasyPure^®^Viral DNA/RNA Kit (TransGen, China) and stored at −80°C for the next step. ZIKV RNA was examined by one-step real-time quantitative reverse transcription PCR (qRT-PCR) using the Mastercycler^®^ ep realplex Real-time PCR System (Eppendorf, Germany). ZIKV RNA copies were calculated based on the standard curve which was determined by plasmid containing specific sequence. The following primers were used: ZIKV-F: 5′-TTGGTCATGATACTGCTGATTGC-3′; ZIKV-R: 5′-CCTTCCACAAAGTCCCTATTGC-3′; ZIKV-probe: 5′-FAM-CGGCATACAGCATCAGGTGCATAGGAG-BHQ1-3′.

### ELISA for Measuring the Concentration of Testosterone and Inhibin B in Mouse Sera

The concentration of testosterone and inhibin B in the sera of BALB/c mice was detected by Mouse Testosterone (ml001948, mlbio, Shanghai, China) and Inhibin B ELISA kit (ml301823, mlbio, Shanghai, China), respectively. According to the manual, 50 μl standard or testing samples were added to a 96-well plate, which was coated with purified mouse testosterone or inhibin B antibody combined with HRP labeling. HRP-conjugate reagent was added to each well, except blank well (no sample; HRP-conjugate reagent added as background). The plate was closed with closure plate membrane and incubated at 37°C for 60 min. After washing, chromogen solution was added and incubated for 15 min at 37°C. Stop solution was added to each well, and absorbance was read at 450 nm.

### Assay to Detect the Inhibitory Activity of Z2 on ZIKV Infection in TM4 Cells

Peptide Z2 was dissolved in DMSO and diluted to different concentration by DMEM/F12. Then 125 μl of different ZIKV strains were incubated with Z2 for 2 h at 37°C. The mixture was added to 5 × 10^4^ cells seeded into a 24-well plate and incubated at 37°C for 2 h. After the culture supernatant was replaced by DMEM/F-12 with 2% horse serum, cells were cultured for 48 h at 37°C. Then the culture supernatant was collected to detect ZIKV RNA copies by qRT-PCR, as described above.

### Assay to Detect Z2-Mediated Inactivation of ZIKV

The ability of Z2 to inactivate different ZIKV strains was determined as follows. Briefly, 100 μl Z2 or Z2-scr, at graded concentration were added to 100 μl ZIKV (5 × 10^3^ PFU/ml), followed by incubation at 37°C for 2 h. Then, PEG-8000 and NaCl were added to the treated virus at final concentration of 10% and 0.67M, respectively. After incubation on ice for 2 h, the mixture was centrifuged at 13,000 rpm for 1 h. The supernatants were removed, and the pellet was resuspended in 200 μl DMEM with 2% FBS. The infectivity of the ZIKV particles in the pellet was determined by CCK-8 on BHK-21 cells or qRT-PCR on TM4 cells.

### Histopathological Analysis

The testis and epididymidis of Z2- or vehicle-treated ZIKV-infected mice and mock-infected mice were all collected post mortem. Tissues were fixed in Bouin’s overnight, dehydrated, embedded in paraffin and sectioned. Then the sections (4 μm thick) were stained by H&E. Subsequently, observation was made *via* panoramic scanner (3D HISTECH Pannoramic MIDI, Hungary).

### Statistical Analysis

Student’s unpaired two-tailed *t*-test was used to monitor the distribution of Z2 in male A129 mouse body and testicular tissue and to analyze the difference of viral RNA level in sera or tissues between Z2- and vehicle-treated A129 mice. One-way ANOVA was used to examine the effect of Z2 on the weight, length and width of testes, as well as sperm count, sperm motility and progressive sperm motility among the three groups. *P*-value was calculated by GraphPad Prism software, v. 7.00, and significant difference was achieved with *P*-value less than 0.05. ^∗^*P* < 0.05; ^∗∗^*P* < 0.01; ^∗∗∗^*P* < 0.001; ^∗∗∗∗^*P* < 0.0001.

## Results

### Z2 Inhibited Infection of ZIKV Strains of Asian and African Lineages in TM4 Cells

To determine the protective effect of Z2 on ZIKV infection of testicular tissue, we tested if Z2 could inhibit infection by different ZIKV strains of Asian and African lineages in mouse Sertoli TM4 cells, which are nurse-like cells that support spermatogenesis (Wei et al., 2018) and important target cells for ZIKV testicular infection. ZIKV SZ01 (Asian lineage), FLR (Asian lineage), or MR766 (African lineage) was pretreated with Z2 at different concentration before addition of TM4 cells and incubation at 37°C for 2 h. After replacement of culture medium and further incubation for 48 h, the viral copies in the supernatant were examined by qRT-PCR. As shown in Figures 1B–D, Z2 treatment resulted in a decrease of ZIKV copies in a dose-dependent manner. Considering that Z2-mediated inhibition of ZIKV infection is possibly attributed to its viral inactivation activity (Yu et al., 2017), different strains of ZIKV were incubated with Z2 at different concentration for 2 h at 37°C, followed by separating virions from the unbound free peptide by PEG 8000 and detecting the infectivity of Z2-treated ZIKV in TM4 cells (Figure 1E) and BHK-21 cells (Supplementary Figure S1). We found that Z2-treated ZIKV strains lost their infectivity in a dose-dependent manner with 50% effective concentration (EC_50_) of 2.74 ± 0.44 μM (for SZ01), 10.21 ± 1.00 μM (for MR766) and 8.96 ± 0.95 μM (for FLR), respectively, suggesting that Z2 inhibits infection of ZIKV strains of both Asian and African lineages in TM4 and BHK-21 cells *via* inactivation of virions.

### Z2 Could Be Distributed in the Testis and Epididymis of Mice

**FIGURE 2:**
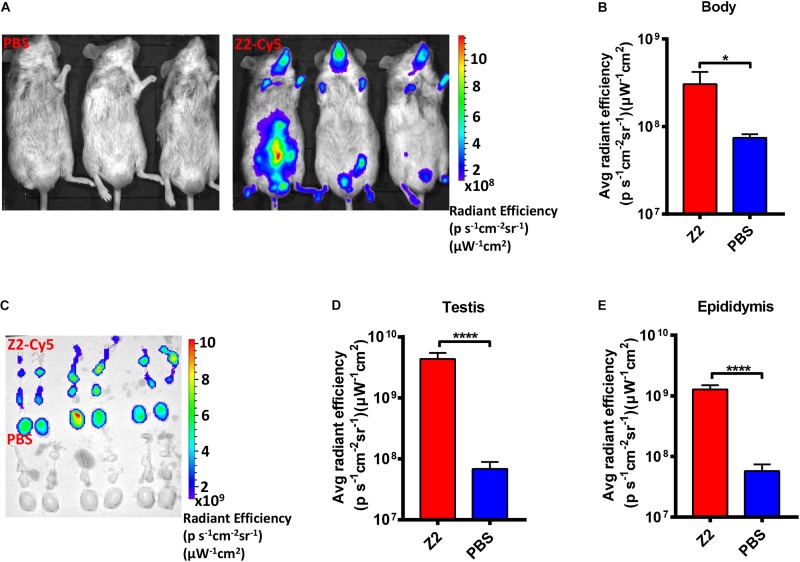
Distribution of Z2 in the testicular tissue of male A129 mice. **(A)** Imaging of male A129 mice treated with Z2-Cy5 or PBS by the IVIS Lumina K Series III from PerkinElmer. Male A129 mice were injected intraperitoneally with 100 μg Z2-Cy5 (*n* = 3) or PBS (*n* = 3) as control, followed by imaging analysis. **(B)** The statistical analysis of fluorescence signal intensity in mouse body. Data were presented as means ± SD. **(C)** Imaging of the testes and epididymides from male A129 mice. **(D)** Statistical analysis of fluorescence signal intensity in testis. Each sample was tested in triplicate and the data were presented as mean ± SD. **(E)** Statistical analysis of fluorescence signal intensity in epididymis. Data were presented as means ± SD. ^∗^*P* < 0.05; ^****^P < 0.0001, Student’s two-tailed *t*-test.

To determine whether Z2 entered testicular tissue of male mice, we employed Cy5-conjugated Z2 peptide (Z2-Cy5) to detect the distribution of Z2 in the organs of male mice. As shown in Figure 2A, the bodies of the Z2-Cy5-treated mice showed a strong fluorescence signal with average radiant efficiency of about 3.05 × 10^8^ (ps^–1^cm^–2^sr^–1^) (μW^–1^cm^–2^), which is significantly higher than that in PBS-treated mice (*P* = 0.0269, Student’s two-tailed *t*-test; Figure 2B). Then, the testes and epididymides were collected for examination of the fluorescence signal in the testicular tissue. As expected, both testes and epididymides of mice treated with Z2-Cy5 showed a strong fluorescence signal, while those in the PBS-treated mice displayed no significant fluorescence signal (Figure 2C). The average radiant efficiency in testes and epididymides of Z2-Cy5-treated mice was significantly higher than that of PBS-treated mice (*P* < 0.0001, Student’s two-tailed *t*-test; Figures 2D,E). These results suggest that Z2 peptide can be distributed in the testis and epididymis of male mice.

### The Protective Effect of Z2 on Male A129 Mice Challenged With ZIKV

**FIGURE 3:**
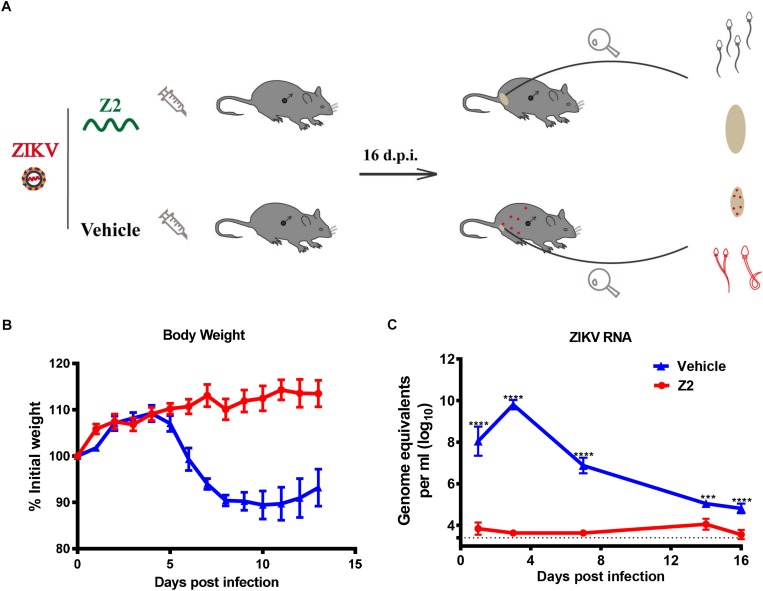
Protective effect of Z2 against ZIKV infection in male A129 mice. **(A)** Schematic diagram of animal experiment. **(B)** The change of mouse body weight was monitored daily for 14 days. **(C)** The change of ZIKV RNA level in mouse sera were detected by qRT-PCR at days 1, 3, 7, 14, and 16 after ZIKV infection. Male A129 mice were intraperitoneally injected with 200 PFU ZIKV with Z2 (15 mg/kg) or vehicle on day 0, followed by daily injection of Z2 (15 mg/kg) or vehicle for six consecutive days. Each sample was tested in triplicate and the data were presented as mean ± SEM, ^∗∗∗^*P* < 0.001; ^****^P < 0.0001, Student’s two-tailed *t*-test.

To determine the protective effect of Z2 against ZIKV infection of male mice, 200 PFU ZIKV were intraperitoneally injected into male A129 mice (type I interferon receptor-deficient). The infected mice were i.p. administered with Z2 at 15 mg/kg of body weight or vehicle, respectively, daily for 7 days (Figure 3A). Mice in the mock-infected group received PBS as normal control. Results showed that the Z2-treated mice had neither weight loss (Figure 3B) nor obvious clinical symptoms, consistent with mice in the mock-infected group (data not shown). However, in the vehicle-treated group, mouse body weight began to decline from the fifth day post-infection (d.p.i.) (Figure 3B), and some symptoms, like hunched posture and ruffled fur, appeared. We then examined viral copies in sera of Z2- or vehicle-treated mice at different time points by qRT-PCR. A high level of viral load was detected in sera of vehicle-treated mice, e.g., about 10^10^ copies/ml at 3 d.p.i. (Figure 3C). However, viral load in sera of Z2-treated mice (Figure 3C) was as low as 10^4^ copies/ml at all time points tested, significantly lower than that of vehicle-treated mice. These results suggest that Z2 can exert protection against ZIKV infection of male mice.

### Z2 Protects Mice Against ZIKV-Induced Damage of Testicular Tissue

**FIGURE 4:**
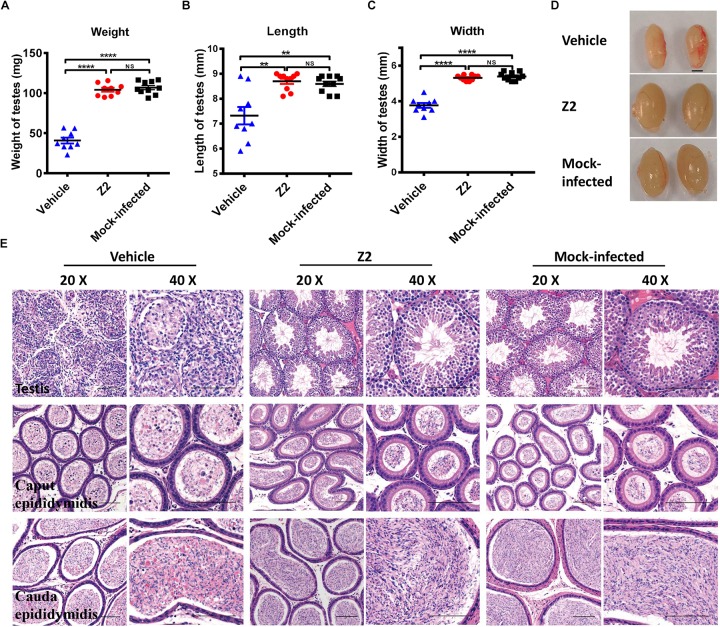
Z2 effectively attenuated damage to testes and epididymides of ZIKV- infected male A129 mice. **(A)** The weight, **(B)** length, and **(C)** width of testes from Z2- or vehicle-treated ZIKV-infected and mock-infected male A129 mice at day 16. Each symbol represents one testis; all horizontal bars indicate mean, and error bars reflect SEM. **(D)** The representative image of testes from Z2- or vehicle-treated ZIKV-infected and mock-infected male A129 mice at day 16. Scale bar, 2 mm. **(E)** Histopathological analyses of testes and epididymides collected from Z2- or vehicle-treated ZIKV- infected male A129 mice and mock-infected mice used as a control. Scale bar: 100 μm. Upper panel, testes; middle panel, caput epididymides; lower panel, cauda epididymides. ^∗∗^*P* < 0.01; ^****^P < 0.0001; one-way analysis of variance with Tukey’s multiple comparison *post hoc* tests.

To further evaluate the protective effect of Z2 against ZIKV-induced damage of testicular tissue in male mice, all mice were sacrificed at 16 d.p.i. and their testes were collected for analysis of weight and size. We found that testis weight in vehicle-treated mice was around 50 mg, which was significantly lower than that in Z2-treated mice (∼100 mg) (*P* < 0.0001, one-way ANOVA, Figure 4A). The length and width of testes in vehicle-treated mice were both significantly decreased compared with those of testes in Z2-treated mice (*P* = 0.0022 and *P* < 0.0001, one-way ANOVA, Figures 4B,C). No significant difference in weight (*P* > 0.5, one-way ANOVA, Figure 4A), as well as length (*P* > 0.5, one-way ANOVA, Figure 4B), and width (*P* > 0.5, one-way ANOVA, Figure 4C) of testes were noted between Z2-treated ZIKV-infected mice and mock-infected mice. The representative image of testes from the three groups of mice were shown in Figure 4D.

Subsequently, we examined the testes and epididymides for histopathological changes. The results of H&E staining of testes in vehicle-treated mice revealed that the normal architecture of the seminiferous tubule was seriously destroyed and replaced with an infiltrate of mixed inflammatory cells and necrotic debris, accompanied by degeneration of the spermatogenic lineage (Figure 4E, upper panel). The connective tissue areas surrounding the seminiferous tubule had also been infiltrated by a large number of inflammatory cells (Figure 4E, upper panel). However, the architecture of the seminiferous tubule in the testes of both Z2-treated and mock-infected mice was intact and clear. Spermatogenic cells at different stages were organized tightly and identified clearly (Figure 4E, upper panel).

Histological analysis of epididymis showed that epididymides from mice in the vehicle-treated group were also damaged. Sperm in the lumen of caput epididymides decreased precipitously, only to be replaced by secretions and numerous necrotic epithelial cells (Figure 4E, middle panel). The lumens of cauda epididymis contained degenerating spermatozoa and a small number of normal spermatozoa, accompanied by scattered necrotic epithelial cells and inflammatory cells (Figure 4E, lower panel). However, histological analysis of the caput epididymis and cauda epididymis showed no apparent microscopic differences between Z2-treated and mock-infected mice (Figure 4E middle, lower panel). The architecture of the caput epididymis and cauda epididymis in these two groups was normal with no obvious morphological damage, suggesting that Z2 protected testicular tissue against ZIKV-induced pathological damage.

### Z2 Protects Mice Against ZIKV-Induced Spermatic Damage

**FIGURE 5:**
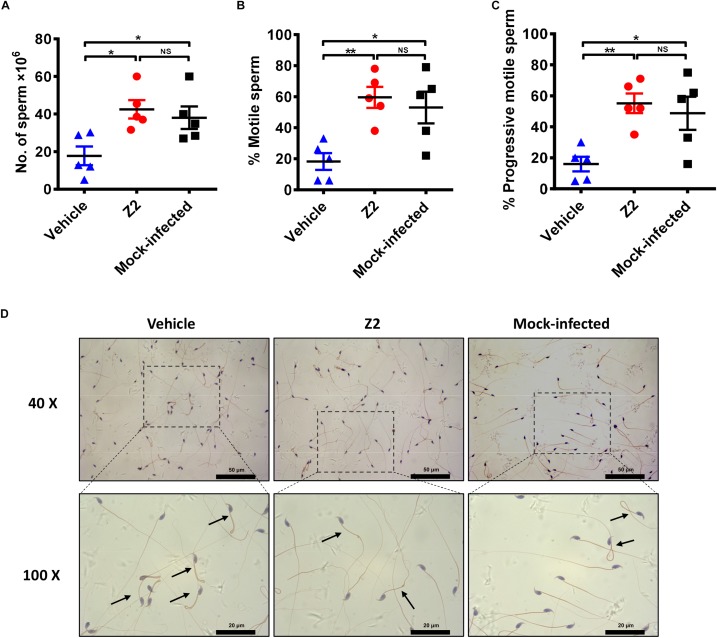
Sperm characterization in Z2-treated ZIKV-infected male A129 mice. **(A)** Sperm count; percentage of **(B)** total motile sperm and **(C)** progressive motile sperm was analyzed by CASA. Each symbol represents data from individual mice; all horizontal bars indicate mean, and error bars reflect SEM. **(D)** Morphological features of sperm from vehicle- (left) or Z2- (middle) treated ZIKV-infected and mock-infected (right) mice. Mature sperm collected from cauda epididymides were stained by Papanicolaou, and the images were taken at 40 and 100× magnification. Black arrows indicated abnormal sperm. ^∗^*P* < 0.05; ^∗∗^*P* < 0.01, one-way analysis of variance with Tukey’s multiple comparison *post hoc* tests.

We used CASA to evaluate the protective effect of Z2 on the count and motility of mouse sperm. As shown in Figure 5, the sperm count of Z2-treated mice was significantly higher than that of the vehicle-treated group (*P* = 0.0124, one-way ANOVA; Figure 5A). Meanwhile, the percentages of total (Figure 5B) and progressively (Figure 5C) motile sperm in Z2-treated mice were dramatically higher than those in the vehicle-treated group (*P* = 0.0055 and *P* = 0.0075, one-way ANOVA), but similar to that in the mock-infected mouse group (*P* > 0.05, one-way ANOVA). Papanicolaou staining of morphological spermatic features revealed more noticeable teratogenesis of sperm in vehicle-treated mice compared to the other groups (Figure 5D).

**FIGURE 6:**
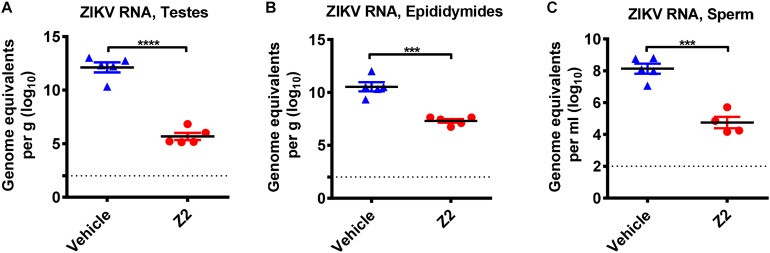
Z2 inhibited ZIKV replication in testes, epididymides and sperm. ZIKV RNA copies in **(A)** testes, **(B)** epididymides, and **(C)** sperm of Z2- or vehicle-treated ZIKV-infected male A129 mice at day 16 were detected by qRT-PCR. Each symbol represents data from individual mice; all horizontal bars indicate mean, and error bars reflect SEM. Experiment was repeated at least twice. ^∗∗∗^*P* < 0.001 or ^****^P < 0.0001 respectively, Student’s two-tailed *t*-test.

To investigate whether the protective effect of Z2 on testicular tissue results from the reduction of local viral load, we examined the viral copies in different testicular tissues. Results showed a high level of viral RNA (10^10^–10^13^ equivalents per g) detected in the testis and epididymis of vehicle-treated mice at 16 d.p.i., much higher than that (10^5^–10^7^ equivalents per g) in Z2-treated mice (Figures 6A,B). Notably, ZIKV RNA was also detected (up to 10^8^ equivalents per ml) in the mature sperm collected from cauda epididymis in vehicle-treated mice, which was significantly higher than that in Z2-treated mice (*P* = 0.0002, Student’s two-tailed *t*-test; Figure 6C).

### Z2 Is Safe for Male BALB/c Mice

**FIGURE 7:**
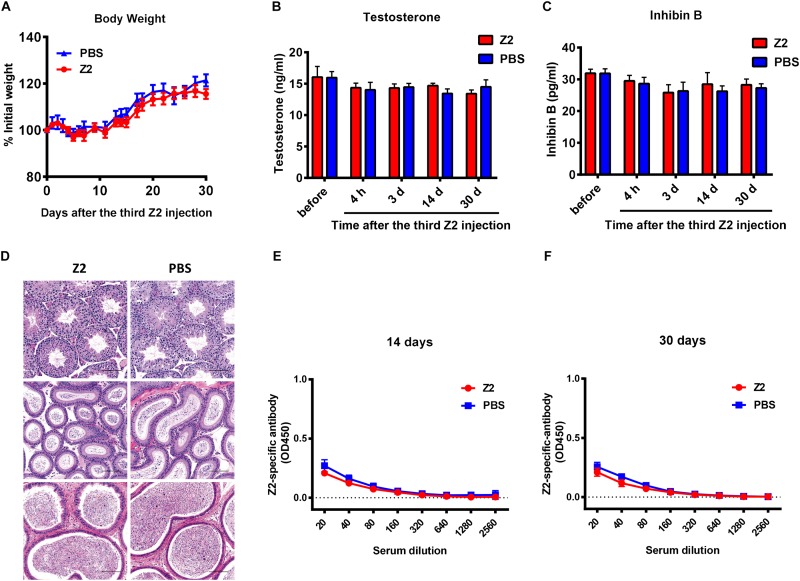
Safety analysis of Z2 for male BALB/c mice. BALB/c mice were injected with Z2 (100 mg/kg/day, i.v.) for 3 days (*n* = 5), and another group of mice (*n* = 5) received PBS as a control. **(A)** Body weight change of BALB/c mice at different time points. Data were presented as means ± SEM. Concentration of **(B)** testosterone and **(C)** inhibin B in sera before and after Z2 injection. Data were presented as means ± SEM of triplicate experiments. **(D)** Histological analysis of the testis and epididymis collected from Z2- or PBS-treated male BALB/c mice. Scale bar: 100 μm. **(E)** Z2-specific antibody response in mice 14 days after i.v. administration of Z2 or PBS. **(F)** Z2-apecific antibody response in mice 30 days after i.v. administration of Z2 or PBS. Each sample was tested in triplicate and the data were presented as mean ± SD.

Finally, to examine the safety of Z2 for male mice, male BALB/c mice were injected intravenously with Z2 at 100 mg/kg of body weight (*n* = 5) or PBS (*n* = 5). Results showed that body weight change of mice was nearly consistent in the two groups (Figure 7A), indicating that Z2 peptide did not cause significant harm to the male mice. Since the levels of testosterone and inhibin B reflect testicular function and sperm count, the concentration of these hormones in mouse sera at the indicated time points was measured. We found no significant difference between the Z2- and PBS-treated groups at all time points tested (Figures 7B,C), suggesting Z2 may not affect the function of testis or sperm. We then compared the potential histopathological changes between the two groups. As shown in Figure 7D, H&E analysis of testis and epididymis revealed no obvious pathological abnormality in mice treated with Z2 compared with the PBS group. Besides, the titer of Z2-specific antibody in sera of mice at 14 and 30 days post-injection was detected. As shown in Figures 7E,F, no significant level of Z2-specific antibody was detected in the sera of mice that were intravenously injected with high doses of Z2 peptide, consistent with the finding from our previous report for studying anti-MERS-CoV peptides (Xia et al., 2019). This result suggests that Z2 peptide consisting of 33 amino acids is unable to elicit a significant Z2-specific antibody response after it is intravenously administered in the absence of adjuvant. Therefore, Z2 is safe for male mice, especially for their testicular tissue.

## Discussion

Currently, many studies have reported the deleterious effects of ZIKV on male testicular tissue, causing severe damage of testis and epididymis, even leading to infertility (Ma et al., 2017; Uraki et al., 2017). Two DNA vaccines were reported to reduce ZIKV persistence in the testicular tissue and ZIKV-associated pathological lesion (Griffin et al., 2017), or partially prevent infertility of male mice (de La Vega et al., 2018). However, no effective and safe antiviral agent has ever been reported to prevent or treat ZIKV infection in testicular tissue. Our previous study has demonstrated that Z2 peptide is highly effective in inhibiting ZIKV infection *in vivo* and *in vitro* (Yu et al., 2017). Noticeably, it can penetrate the placental barrier to inhibit vertical transmission of ZIKV in pregnant mice. However, whether Z2 could cross the BTB and protect testicular tissue against ZIKV infection remained unknown.

Several studies have reported that Sertoli cells play an important role in the entry of ZIKV into the seminiferous tubules and support long-term replication of ZIKV in the testicular tissue (Siemann et al., 2017; Kumar et al., 2018). We found that Z2 peptide possesses potent antiviral activity against ZIKV infection in BHK-21 and Vero cells (Yu et al., 2017). In this study, we found that Z2 was also highly effective in inhibiting infection of divergent ZIKV strains with Asian and African lineages in TM4 cells, the mouse Sertoli cell line. Particularly, Z2 treatment *via* intraperitoneal injection resulted in dramatically decreased ZIKV RNA level in the testis of A129 mice, suggesting that Z2 can protect testis against ZIKV infection in Sertoli cells.

Meanwhile, we employed Z2-Cy5 to examine whether Z2 could enter seminiferous tubule, and we found that intraperitoneally injected Z2 could be distributed in the testicular tissue of male A129 mice, consistent with the observation in mice intravenously administered with Z2 (Yu et al., 2017). However, because of the intricate structure of capillary vessel and seminiferous tubule in mouse testis, we could not obtain sufficient evidence to prove that Z2 crossed BTB into seminiferous tubule. H&E analysis showed no obvious pathological damage in the testicular tissue of Z2-treated mice, but it did reveal severe pathological damage in the testis and epididymis of vehicle-treated mice, consistent with the findings of other studies (Govero et al., 2016; Ma et al., 2017). When combined with evidence that viral load in mature sperm of Z2-treated A129 mice was significantly decreased, we speculate that Z2 may, indeed, cross the BTB and enter seminiferous tubule to inhibit ZIKV infection in the sperm.

Zika virus infection in the testicular tissue not only damages male testicular tissue, resulting in pathological lesion of testes and epididymides, but also produces ZIKV-infected semen, causing infertility. In addition, ZIKV in semen of an infected male can be sexually transmitted to his pregnant partner (Russell et al., 2017; Nelson et al., 2018), who can further pass the virus to her fetus, causing congenital Zika syndrome in the newborn (Yarrington et al., 2019). Sexual transmission may also contribute to the spread of ZIKV in regions where the *Aedes mosquito* is not endemic (Rowland et al., 2016). Here we found that Z2 treatment could significantly reduce viral load in sperm of ZIKV-infected A129 mice and improve the number and motility of sperm, implying that application of Z2 can limit the damage to testicular tissue and sperm caused by ZIKV infection and reduce the risk of sexual transmission of ZIKV.

## Conclusion

Z2 administered *via* intraperitoneal or intravenous injection could be distributed in mouse testicular tissue, protect the tissue against ZIKV infection and ZIKV-induced pathological damage and poor sperm quality, suggesting that Z2 peptide has the potential to be further developed as an anti-ZIKV therapeutic for treatment of ZIKV infection and attenuation of ZIKV-induced damage in the testicular tissue.

## Data Availability Statement

All datasets generated for this study are included in the manuscript/Supplementary Files.

## Ethics Statement

The animal study was reviewed and approved by Shanghai Public Health Clinical Center Animal Welfare and Ethics Committee Institutional Laboratory Animal Care and Use Committee at Fudan University.

## Author Contributions

LL, SJ, and JH conceived and designed the experiments. LS, YM, PZ, QW, and WX performed the experiments. FT, YW, MX, and WL carried out the CASA and H&E staining analysis about A129 mice. LS and YM analyzed the data. LL, SJ, JH, and LS wrote the manuscript.

## Conflict of Interest

The authors declare that the research was conducted in the absence of any commercial or financial relationships that could be construed as a potential conflict of interest.

## Footnotes

1 https://www.who.int/emergencies/diseases/zika/epidemiology-update/en/
