# Supplementary material for: A Peptide-Based Virus Inactivator Protects Male Mice Against Zika Virus-Induced Damage of Testicular Tissue
Source: Front Microbiol. 2019 Sep 27;10:2250. doi: 10.3389/fmicb.2019.02250 (PMC6777420; doi:10.3389/fmicb.2019.02250)
Supplement: Supplementary file 1 [file Data_Sheet_1.PDF]

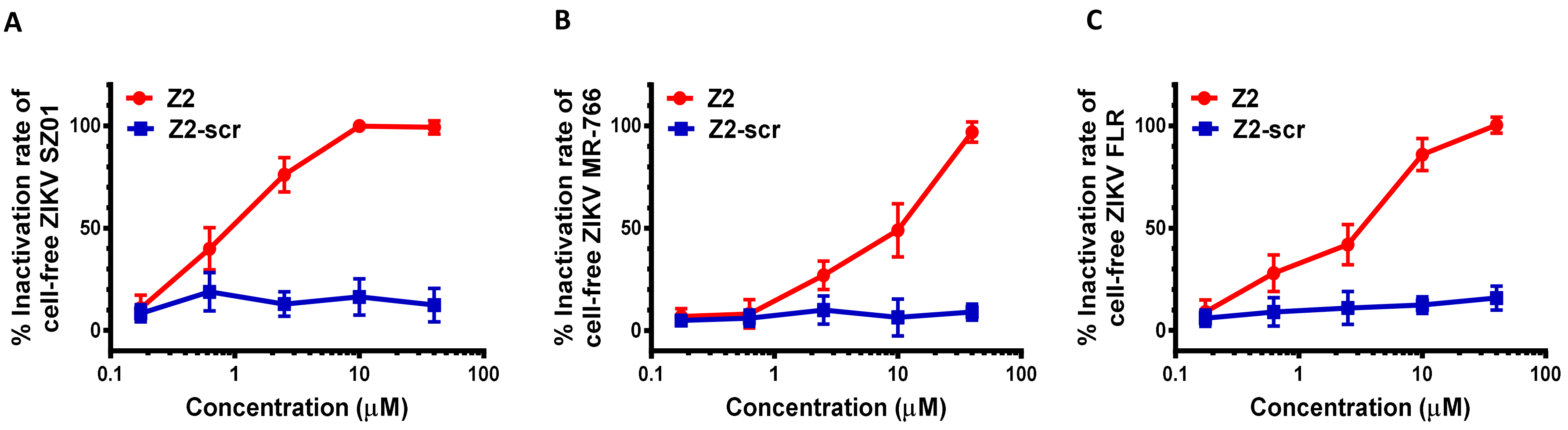

FIGURE S1| Z2 inactivated ZIKV strains with Asian and African lineages tested in BHK-21 cells. Z2-treated ZIKV strain SZ01 **(A)**, MR766 **(B)** and FLR **(C)** lost infectivity on BHK-21 cells irreversibly. After incubation with Z2 or Z2-scr at 37°C for 2 h, ZIKV particles were separated from the unbounded Z2 by PEG 8000 to measure their infectivity on BHK-21 cells. Data were presented as means ± SD. The error bars represent data from two independent experiments.
